# Introducing HPV Vaccine in Developing Countries - Addressing the Challenge

**DOI:** 10.4103/0970-0218.58407

**Published:** 2009-10

**Authors:** Prianka Mukhopadhyay, Bhaskar Paul

**Affiliations:** Department of Community Medicine, NRS Medical College, Kolkata, India; 1 Department of Gynaecology & Obstetrics, Medical College, Kolkata, India. E-mail: docprianka@yahoo.co.in

Sir,

Vaccines have played a crucial role in preventing infectious disease-associated morbidity and mortality, worldwide. Vaccinations have achieved several milestones e.g. eradication of smallpox, imminent eradication of polio, and a dramatic reduction in the prevalence of childhood infections like diphtheria, tetanus and pertusis. Many newer, safe and effective vaccines, like those against Hib, Pneumococcus, Hep A, Chicken pox, Rotavirus, HPV to name a few, are only now making an entry into the market of developing countries, though they were introduced decades earlier in the developed countries.(1) Current findings based on results from clinical trials indicate that a three-dose schedule of both bivalent and quadrivalent vaccine formulations against HPV are highly immunogenic with sero-conversion rates to all targeted HPV types of over 98%.(2) However, the mere availability of an effective vaccine is not synonymous with an effective vaccination program.

Challenges to the introduction of HPV vaccine in our country:

- Relatively high cost of HPV vaccine.(3)
- How to reach the target population? Coverage with existing EPI vaccines is only 43.5% for fully vaccinated children aged 12-23 months with an estimated 12.5 million under-immunized children each year.(4)
- Poor healthcare infrastructure, inadequate maintenance of cold chain and injection safety.
- Limited resources of UIP and competition faced from other newer vaccines like Hib, pneumococcal, rotavirus, meningococcal, JE vaccines etc.(5)
- Poor success of secondary prevention methods e.g. HPV screening, Pap testing, or visual inspection in the country.
- Religious barriers, cultural taboos and misconceptions influencing acceptance by doctors, parents, adolescents and public in general e.g. a vaccine targeted primarily to females and associated with a sexually transmitted infection may exacerbate rumors like a plot to sterilize young girls.(5)
- Lack of political will.

Without a doubt, one of the greatest barriers to the introduction of this vaccine is its price.(6) Several mechanisms to make vaccines affordable in the context of developing countries have been proposed, like differential pricing, advance market commitments (AMC) and voluntary and compulsory licensing. The most prominent model is differential (or tiered) pricing, where the patent owner retains production and control of pricing between segmented markets.(7) Proposals based on advanced market commitment provides an assured price subsidy for developing country purchase of a future vaccine meeting predefined standards. This mechanism can provide the industry with greater assurances of earning a reasonable return on their investment and act as a motivation to serve the poorest developing countries.(8) Differential pricing may be combined with donor financing support and advance market commitments. A new type of license that uses market forces to lower prices through generic competition in low and middle-income countries while ensuring that pharmaceutical companies are appropriately reimbursed for their research and development holds much promise.(7) Wider global availability of affordable vaccines can be achieved through building local or regional clinical trial and vaccine production capacity.(1) Hepatitis B vaccine production in South Korea was a major factor in bringing the price of this vaccine to affordable levels in the developing world.(5)

Countries can use the established immunization networks or networks of Sexual and Reproductive Health (SRH) services such as family planning, pre- and post-natal care or a mixture of systems to deliver the vaccine to the target population. Immunization service delivery is the most successful public health system in the world and the infrastructure of trained staff, cold chain and logistics, clinics and outreach services, and information systems is a resource that could be utilized to deliver HPV vaccine.(5) The immunization system will need to be substantially enlarged to reach large numbers of adolescents and women of child-bearing age outside the ambit of pre-natal services.(5) Local logistical hurdles must be overcome and the opportunity of strengthening health care infrastructure through vaccination must be utilized.(1)

SRH services could simultaneously provide important contacts for health education and advocacy for HPV and dispel myths and misconceptions regarding the vaccination. Ultimately, the decision of whether and when a vaccine will be introduced will depend on individual countries. To prepare for decisions on HPV vaccine use, the sexual and reproductive health (SRH; including adolescent health), immunization, and cancer control communities need to work together to analyze the appropriate data and build international and national consensus.(5) Local leaders must be convinced of the benefits of vaccination to make immunization a high priority.

Finally, ‘translational research’ can provide the answer to rational introduction of newer vaccines like HPV in developing countries for optimum utilization of available resources.(9)
